# The impact of hip fracture on resilience in health-related quality of life: a cohort study

**DOI:** 10.1007/s41999-025-01213-z

**Published:** 2025-04-29

**Authors:** Stina Ek, Mozhu Ding, Margareta Hedström, Stefan Fors, Karin Modig

**Affiliations:** 1 Unit of Epidemiology, Institute of Environmental Medicine, Karolinska Institutet, Nobels väg 13, S-17165 Stockholm, Sweden; 2 Department of Clinical Science, Intervention and Technology (CLINTEC), Karolinska Institutet, Stockholm, Sweden; 3 Trauma and Reparative Medicine Theme (TRM), Karolinska University Hospital, Stockholm, Sweden; 4 Aging Research Center, Karolinska Institutet & Stockholm University, Stockholm, Sweden; 5 Centre for Epidemiology and Community Medicine, Region Stockholm, Stockholm, Sweden; 6 Department of Public Health Science, Stockholm University, Stockholm, Sweden

**Keywords:** RIKSHÖFT, Fall injuries, Register-based, EQ5D

## Abstract

**Purpose:**

Hip fractures significantly impact health-related quality of life (HRQoL) in older adults. This study aimed to investigate and quantify changes in different domains of HRQoL following a hip fracture and to identify factors associated with maintaining HRQoL (resilience).

**Methods:**

The study included a cohort of 2,529 individuals aged 65 + who experienced their first hip fracture between 2016 and 2020. HRQoL was assessed using the EQ5D-5L tool both prior to and 4 months after the fracture. Resilience was defined as maintaining or improving pre-fracture EQ5D levels at follow-up. Associations between sociodemographic characteristics, acute conditions, medical factors, and resilience in specific EQ5D domains—Mobility, Usual Activities, Self-Care, Pain/Discomfort, and Anxiety/Depression—were analyzed using regression models.

**Results:**

Sixty-nine percent were women and the mean age was 82.6 years. A decline was observed across all EQ5D domains and in the global EQ5D index following a hip fracture. The proportion of patients exhibiting resilience varied by domain, ranging from 36 to 77%, with Mobility being the most adversely affected and Anxiety/Depression the least affected. Factors associated with resilience differed by domain. Mobility, Usual Activities, and Self-Care followed a similar pattern, while Pain/Discomfort and Anxiety/Depression showed distinct patterns. Notably, the absence of fall-risk-increasing medications was the only factor consistently associated with resilience across all domains.

**Conclusions:**

While hip fractures generally lead to declines in HRQoL, a significant proportion of patients demonstrate resilience within 4 months post-fracture. Patterns of resilience vary across EQ5D domains, with physical functions being most impacted and mental health the least.

**Supplementary Information:**

The online version contains supplementary material available at 10.1007/s41999-025-01213-z.

## Key summary points

**Aim:**

To investigate how quality of life, measured with the EQ5D domains, changes after a hip fracture and identify factors that contribute to better recovery in older adults.

**Findings:**

The proportion of patients exhibiting resilience varied by domain, ranging from 36% to 77%, with Mobility being the most adversely affected and Anxiety/Depression the least affected. Factors associated with resilience differed by domain and absence of fall-risk-increasing medications was the only factor consistently associated with resilience across all domains.

**Message:**

Different aspects of HRQoL are affected to different degrees, but targeting safe medication use could help improve recovery and quality of life for people after a hip fracture.

**Supplementary Information:**

The online version contains supplementary material available at 10.1007/s41999-025-01213-z.

## Introduction

A key aspect of healthy aging is the ability to cope with adverse events as they arise—to be resilient. Physical resilience is defined by Whitson et al. [1] as the ability to withstand or recover from functional decline following an acute or chronic health stressor. Hip fractures are an example of such acute stressors. For older adults, a hip fracture can be devastating, often resulting in long-term disability, increased care need, and premature death [2]. In addition, hip fractures also impact wellbeing and quality of life (QoL) [3, 4].

Good QoL is an essential element of healthy aging. Although health-related quality of life (HRQoL) correlates with objective health [5], HRQoL is not fully explained by objective health, as changes in health and function affect people differently depending on their lifestyle [6–8]. It is important to pay attention also to life quality to enable person-centered work methods in healthcare and public health work.

Previous research has shown that HRQoL decreases after a hip fracture, and that the decrease is influenced by pre-fracture mobility, polypharmacy, depression and anxiety, independent living, and independence in activities of daily living [9, 10]. However, no previous studies have explored how common it is to *maintain* HRQoL after a hip fracture, in contrast to studying the decrease in HRQoL, and what characterizes individuals that manage to be resilient in their HRQoL after a hip fracture.

A commonly used tool to measure HRQoL among older adults is the European Quality of Life —five dimensions (EQ5D-5L). EQ5D is a standardized and validated self-assessed instrument that aims to measure different dimensions of HRQoL: mobility, self-care, usual activities, pain/discomfort, and anxiety/depression [11–15]. EQ5D is tested and validated in the Swedish hip fracture population with good results [15–17]. Most previous studies have focused on the combined effect of the domains and only one previous study by Gjertsen et al. has investigated how each domain is affected after a hip fracture [18]. The findings showed that even if all domains worsened after a hip fracture, the patterns differed across domains. From a secondary prevention perspective, it is important to understand which domains are most affected and the factors associated with maintaining HRQoL. Therefore, this study aimed to examine the impact of a hip fracture on each EQ5D domain—mobility, self-care, usual activities, pain/discomfort, and anxiety/depression. Additionally, it explored the proportion of hip fracture patients who maintained or improved their HRQoL within 4 months after a hip fracture and the characteristics of these resilient individuals.

## Methods

The study population consisted of individuals aged 65 years and older, who had endured a first hip fracture between 2016 and 2020 and was registered in the Swedish National Hip Fracture Register (SHR), both at baseline and 4 month follow-up. Since the EQ5D battery is optional for hospitals to include in the SHR, the study population is a subsample of all hip fractures who had data on EQ5D recorded during the study period. 4282 individuals performed the EQ5D-5L questionnaire in SHR at baseline, 562 died before the 4 months follow-up, and 1191 did not have follow-up information (either did not conduct the follow-up at all or had missing EQ5D data), resulting in a study population of 2529 individuals. A comparison of baseline characteristics between the study population, individuals who died before follow-up, and individuals with missing follow-up data is presented in Appendix Table 1. Individual level data from SHR were linked to other national registers via the Swedish Personal Identification Number, and thereafter, the data were pseudonymized. The baseline measurement in SHR was gathered in conjunction with the hospitalization for the hip fracture, but the patients are asked to recall their status the week prior to the hip fracture. Therefore, the baseline measurement will be referred to as “pre-fracture” status.

**Table 1:** Baseline characteristics and global EQ5D score, pre- and post-hip fracture (EQ5D index score of 1 = optimal, 0 = worse possible)

	Baseline characteristics	EQ5D index pre-fracture	EQ5D index post-fracture	Statistical difference (t test)
	All, n = 2,529	0.72	0.60	*
Sex				
Women	1752 (69.3)	0.73	0.61	*
Men	777 (30.7)	0.70	0.56	*
Age				
65–79 years	860 (34.0)	0.76	0.66	*
80–89 years	1171 (46.3)	0.71	0.58	*
90 + years	498 (19.7)	0.65	0.52	*
Cohabitation				
Co-living	931 (36.8)	0.78	0.66	*
Living alone	1129 (44.6)	0.77	0.64	*
Care home	469 (18.5)	0.46	0.37	*
Education				
Primary	1213 (48.0)	0.71	0.59	*
Secondary	882 (34.9)	0.72	0.60	*
University	434 (17.2)	0.73	0.62	*
ASA score				
1–2	1147 (45.4)	0.79	0.69	*
3–5	1379 (54.6)	0.66	0.52	*
Fracture type				
Intracapsular	1384 (54.7)	0.72	0.61	*
Pertrochanteric	937 (37.1)	0.72	0.57	*
Subtrochanteric	208 (8.2)	0.70	0.58	*
Surgery method				
Less invasive	870 (34.4)	0.72	0.58	*
More invasive	1659 (65.6)	0.72	0.60	*
Medications				
FRIDs 0	360 (14.2)	0.82	0.71	*
FRIDs 1–2	922 (36.5)	0.76	0.63	*
FRIDs 3 +	1247 (49.3)	0.66	0.54	*
Dementia	377 (14.9)	0.49	0.38	*
Depression	106 (4.2)	0.62	0.47	*
Walking ability, pre-fracture				
High	1582 (62.5)	0.82	0.71	*
Intermediate	808 (32.0)	0.59	0.44	*
Low	139 (5.5)	0.30	0.26	*

The highest attained level of education (primary school, secondary school, or university) was derived from the longitudinal integrated database for health insurance and labor market studies (LISA). Information about cohabitation, fracture type, surgery method used, walking ability, and co-morbidity was derived from SHR. Comorbidity was measured with the American Society of Anesthesiologists physical status classification system (ASA score). SHR is based on interviews with the patient or with a proxy, for example a next-of-kin or caretaker, if the patient is unable to answer. Cohabitation was categorized as Living with someone, Living alone, and living in Care Home. The type of fracture was categorized as intracapsular (including basocervical), pertrochanteric, or subtrochanteric. The surgery method used was divided into “More invasive”, including any type of hip replacement and intramedullary nail and all other surgery methods were categorized “Less invasive”. ASA score was divided into score 1–2 and 3–5, where category 1–2 represents good overall health prior to surgery and 3–5 represents poorer health, a value of 3 represents “A patient with severe systemic disease”. Walking ability was categorized into three: “High” (walking independently), “Intermediate” (walking with support outside or independent inside), and “Low” (walking with support inside or not walking at all). Support could mean either from a stick or frame or from another person. For the items walking ability, use of walking aid, and EQ5D, the patients were asked to answer regarding the week prior to the hip fracture. Diagnosis of depression was extracted from the patient register (inpatient and specialized outpatient care) within 5 years prior to the hip fracture. Dementia was based on diagnosis of dementia in the patient register within 5 years and/or if the hip fracture patient had a known diagnosed dementia registered in SHR. Since dementia is generally underdiagnosed in the patient register, most dementia cases are detected from the SHR. A selection of medications known for their fall-risk-increasing impact for older adults, Fall Risk Increasing Drugs (FRIDs) [19], was extracted from the Prescribed Drugs Register (PDR), and categorized into 0, 1–2, or 3 + number of FRIDs prescribed within a year from the hip fracture.

EQ5D was presented in two different ways, EQ5D scores and EQ5D Index. The score is the value for each domain, where 1 represents the best and 5 the worse value. The EQ5D Index is a weighted global score for all domains with a value between 0 and 1. In the EQ5D Index, a high score is better, 1 is the best, and 0 is the worse [20]. Maintenance or improvement (resilience) of HRQoL was defined as keeping or improving the pre-fracture score after 4 months.

### Statistical analysis

The characteristics of the study population and EQ5D scores per domain were reported as proportions (%). EQ5D score pre- and post-fracture on an individual level was presented in alluvial plots. EQ5D Index, pre-fracture and post-fracture, was presented as means and t tests were used to test statistical change between the two measures in time. Since individuals who reported poorly (value 4 and 5) pre-fracture had a high chance of maintaining their EQ5D level, not due to resilience but due to a floor effect, only individuals with score 1–3 was included when creating the categories “Resilient” and “Non-resilient”. Correlation between the different domains in regards of resilience of HRQoL was tested. Associations between the separate domains and chosen sociodemographic-, acute/hip fracture related-, and medical related factors known to have an impact on prognosis after a hip fracture [21–24] was tested with logistic regressions, with results presented as odds ratios (95% confidence interval), with a first model unadjusted and one model mutually adjusted for all exposures. For each exposure, the category with the highest proportion of the study population served as reference. Since the same individual could score high in a few domains and low in others, the study population differed for each domain and the logistic regressions were conducted separately for each domain, a flowchart of the study population can be found in the supplementary material (Appendix Fig. 1).

This study is conducted in compliance with the ethical rules stated in the Declaration of Helsinki and has obtained ethical approvals from the Stockholm Regional Ethical Review Board (Dnr 2011/136–31/5, 2017/1088–31, 2018/84–32, and 2022–03486-02). SHR uses the opt-out method, meaning that patient consent is not needed to be part of the quality registry, but answering the questionnaires is voluntary. Patients may, however, opt out from inclusion in the registry at any time.

## Results

Table 1 shows the pre-fracture characteristics of the study population. The population consisted of 69% women, with a mean age of 82.6 years (SD = 7.5). A large proportion (45%) lived alone. More than half of the participants (55%) had an ASA score of 3 or higher, indicating severe systemic disease, and 40% were prescribed three or more FRIDs. 13% died before follow-up and these patients were on average older, had more comorbidities and FRIDs, and resided in a care homes to a higher extent (Appendix Table 1 and 2). Participants with missing follow-up data were similar to the overall study population regarding baseline characteristics.

**Table 2:** Proportion resilient (i.e., no loss) in each EQ5D domain, %

	Mobility *n* = 2278	Self-care *n* = 2212	Usual activity *n* = 1959	Pain/Discomfort *n* = 2263	Anxiety/Depression *n* = 2358
All	36.3	57.7	42.4	61.9	77.1
Sex					
Women	37.2	60.5	43.3	63.3	76.1
Men	34.1	51.1	40.2	58.7	79.2
Age					
65–79	39.7	69.2	50.0	59.8	78.4
80–89	34.3	53.5	39.3	61.8	75.9
90 +	34.9	45.6	34.1	65.4	77.5
Education					
Primary	36.8	53.3	39.5	61.3	77.1
Secondary	37.5	60.5	44.7	63.2	76.8
University	32.4	64.2	46.2	60.9	77.7
Living status					
Cohabiting	38.4	62.3	45.7	59.7	77.3
Alone	34.9	58.4	40.6	59.7	76.8
Care home	35.0	38.7	36.1	71.6	77.2
ASA score					
1–2	38.2	66.3	47.5	61.3	78.8
3–5	34.5	49.7	37.4	62.4	75.5
Dementia	34.6	34.5	31.0	71.1	74.5
Walking ability					
High	37.8	67.0	46.1	60.5	78.4
Intermediate	33.4	35.8	31.1	62.7	73.4
Low	27.8	37.1	20.8	74.3	82.1

### Global EQ5D

The mean pre-fracture EQ5D Index was 0.72 (1.0 represents best health). Being female, younger, living independently (either alone or with others), low co-morbidity burden, and high walking ability were associated with higher pre-fracture EQ5D Index.

At the 4-month follow-up, the overall mean EQ5D Index had declined to 0.60. The greatest reductions in EQ5D scores were observed among men, individuals aged 90 years or older, those residing in care homes, and those with intermediate walking ability at baseline (Table 1).

### EQ5D domains

**Fig. 1:**
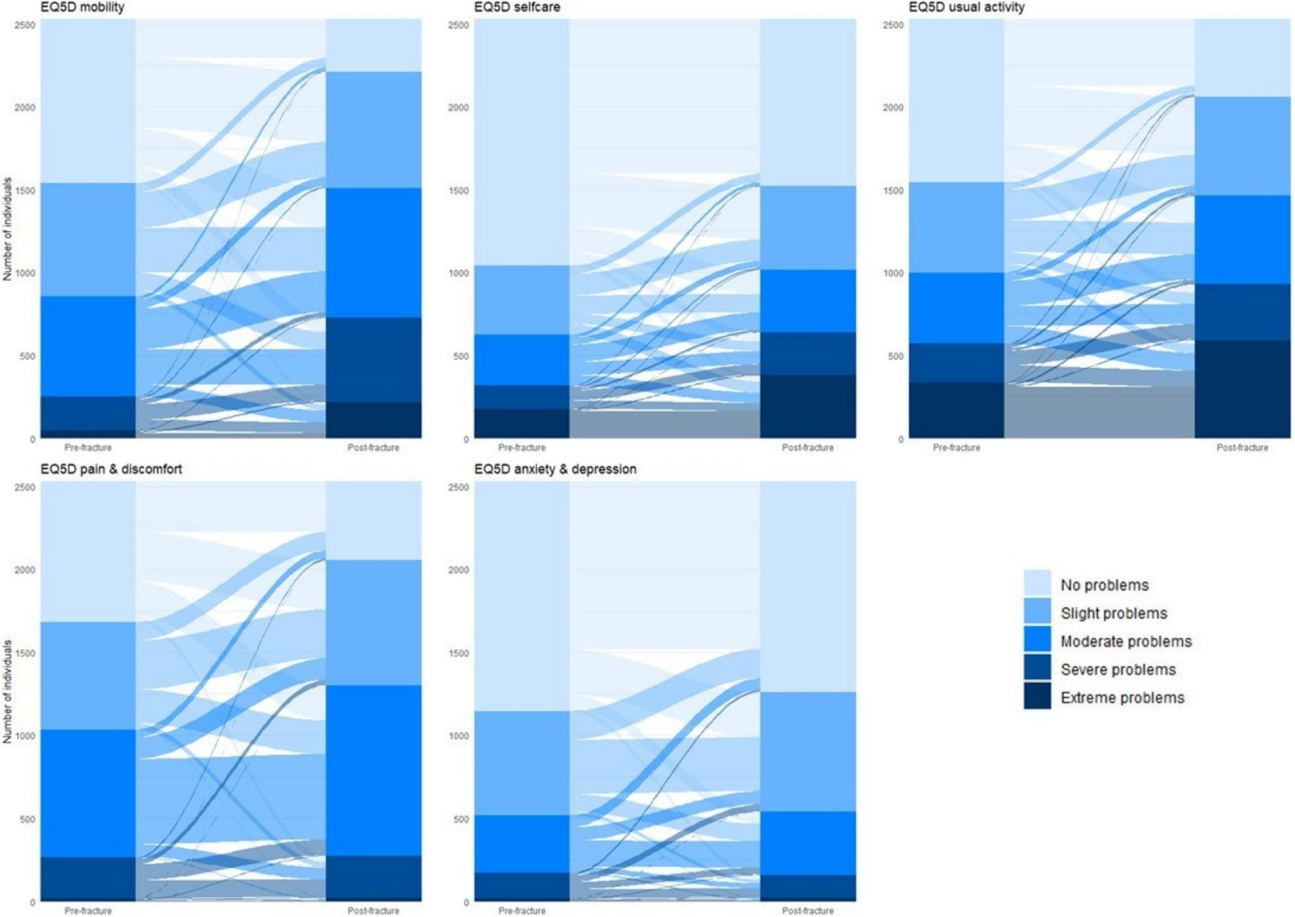
Change in EQ5D domain values pre- and post-fracture per individual

**Fig. 2:**
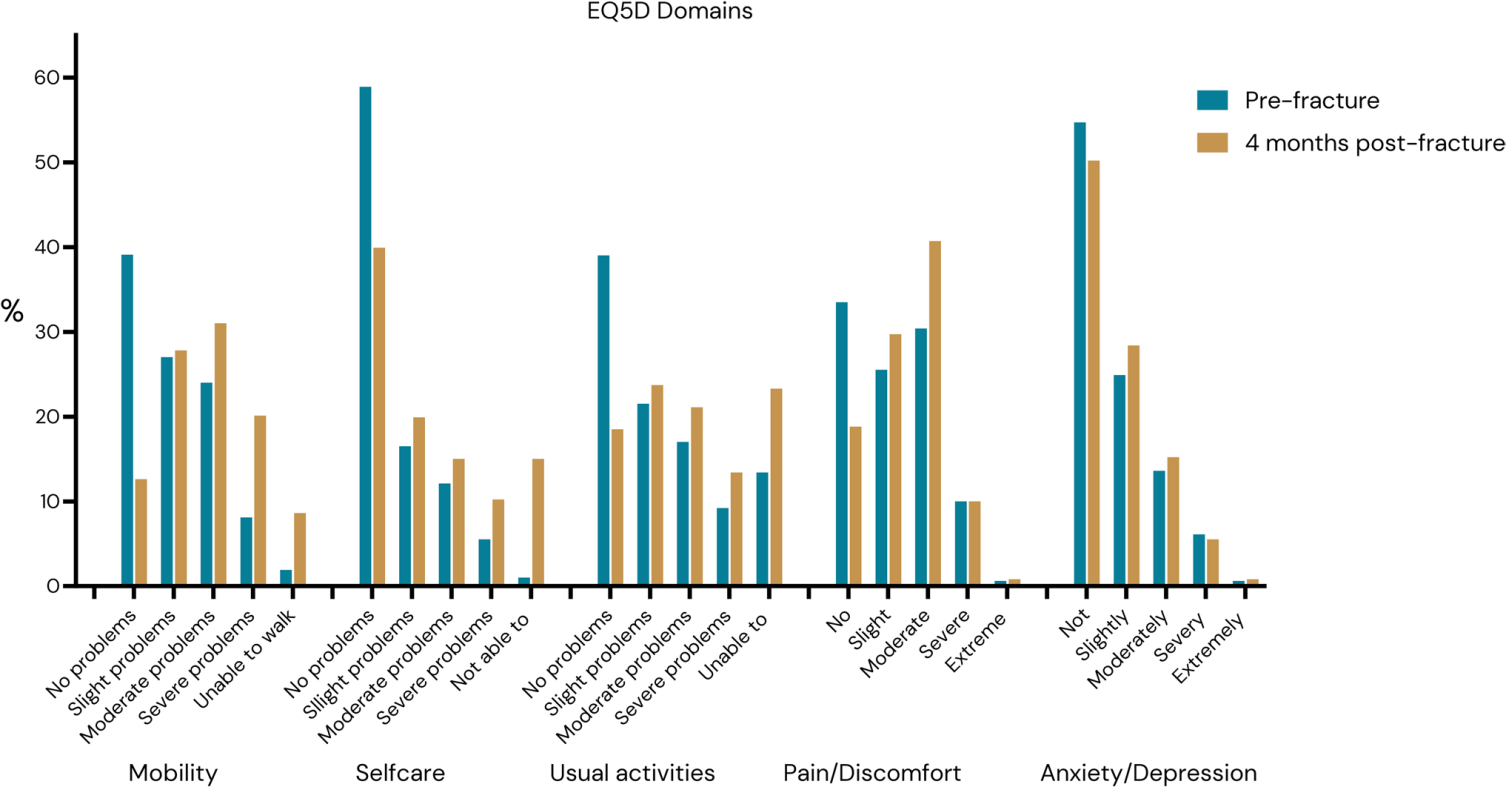
Distribution of the EQ5D domains pre- and post-fracture

**Fig. 3:**
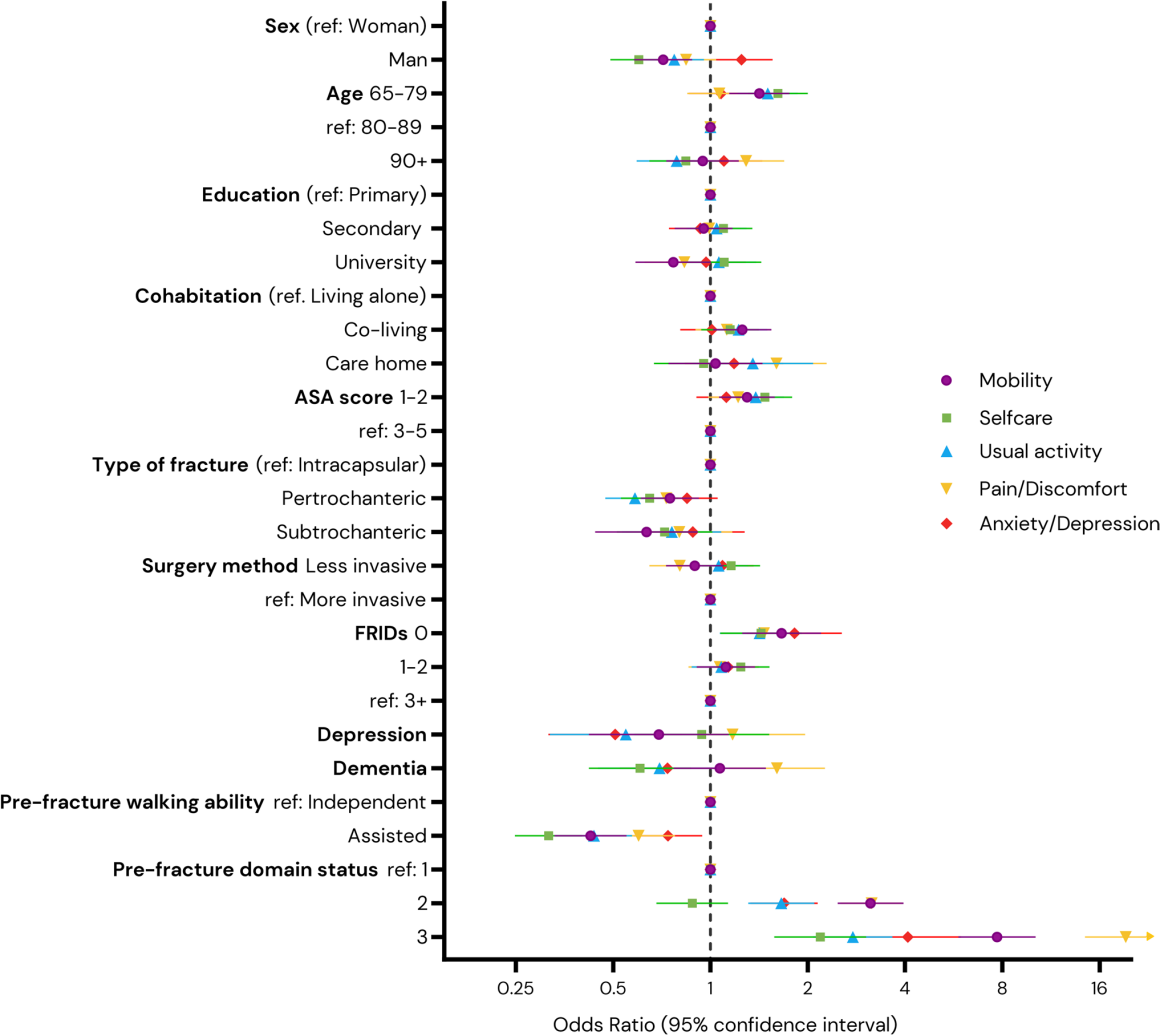
Association between sociodemographic, fracture related- and medical factors and maintaining EQ5D level 4 months after a hip fracture, per EQ5D domain. Mutually adjusted for all exposures (specific baseline level EQ5D per domain)

Figure 1 shows change in the different EQ5D domains pre- and post-fracture. The plot shows that a small part of the resilient individuals even improved their values 4 months after the hip fracture; this is especially evident in the domains pain and discomfort and anxiety and depression. Figure 2 illustrates the distribution of values across each EQ5D domain before and after the hip fracture. The proportion of individuals demonstrating resilience in each domain, categorized by subgroup, is provided in Table 2. The adjusted logistic regression analysis results for each EQ5D domain are shown in Fig. 3 (unadjusted results in Appendix Fig. 3).

### Mobility

39% of participants reported no walking problems before the hip fracture, and 10% had severe mobility restrictions or were unable to walk. Four months post-fracture, the proportion of individuals with no walking problems dropped to 13%, and more than 25% reported severe mobility restrictions or were unable to walk (Fig. 2). Among those who initially had good-to-moderate mobility, only 36% retained their mobility within 4 months after the fracture (Table 2).

Factors positively associated with maintaining mobility included being younger age, cohabiting, having a lower ASA score, not taking any FRIDs, and having a worse baseline mobility score. In contrast, being male, having an extracapsular fracture, and impaired mobility prior to the fracture were negatively associated with maintaining mobility (Fig. 3).

### Self-care

Before the hip fracture, more than half of the participants (59%) reported no difficulties with self-care, and only 1% were unable to wash themselves or get dressed. After the hip fracture, 40% of individuals continued to report no difficulties, while the remaining participants were evenly distributed between experiencing slight problems and being unable to perform self-care tasks such as washing and dressing (Fig. 2). Approximately 58% of participants maintained their EQ5D level in the self-care domain (Table 2).

Factors associated with maintaining self-care levels mirrored those found in the mobility domain, with the notable exception that a diagnosis of dementia was negatively associated with self-care maintenance. Additionally, the pre-fracture self-care level was less significant in influencing this association (Fig. 3).

### Usual activities

Although most individuals were independent in their usual activities before the hip fracture (39%), the variation was larger than for the other EQ5D domains. Notably, 13% of participants were already unable to perform their usual activities prior to the fracture. Four months after the hip fracture, only 19% of individuals retained their independence and 23% were not able to perform their usual activities (Fig. 2). Overall, 42% of participants maintained their usual activity level after 4 months (Table 2).

Similar to the findings in the mobility and self-care domains, factors associated with maintaining the usual activity level 4 months after the hip fracture included younger age, a low ASA score, fewer FRIDS, and higher pre-fracture activity level. Conversely, being male, having a pertrochanteric fracture, a prior diagnosis of depression, requiring assisted walking before the fracture, and having lower pre-fracture activity level was negatively associated with maintaining the prior activity level (Fig. 3).

### Pain and discomfort

Before the hip fracture, 34% of participants reported no pain or discomfort, and less than 1% experienced extreme pain. Four months post-fracture 81% reported some degree of pain. 19% reported no pain or discomfort; however, the change was primarily from no pain to moderate pain, with the proportions of individuals reporting severe or extreme pain remaining unchanged (Fig. 2). A majority reported similar levels of pain before and after the hip fracture (Table 2).

The pattern of pain and discomfort differed from the previously mentioned domains. Factors positively associated with maintaining pain levels included living in a care home, not taking FRIDs, having a dementia diagnosis, and pre-fracture pain levels. In contrast, having a pertrochanteric fracture and impaired walking ability prior to the fracture were negatively associated with the maintenance of pain levels (Fig. 3).

### Anxiety and depression

Before the hip fracture, more than half of the participants (55%) reported no anxiety or depressive symptoms, and very few reported severe problems. Four months post-fracture, there was only a slight increase in the proportion of individuals reporting mild-to-moderate problems, while the rates of those reporting severe-to-extreme problems remained stable (Fig. 2). This domain exhibited the highest level of resilience, with 77% of participants maintaining their EQ5D levels (Table 2).

Factors positively associated with maintaining the anxiety and depression domain level included not taking FRIDs and pre-fracture anxiety/depression status. Conversely, pre-fracture depression and requiring assisted walking were negatively associated with maintaining the domain level (Fig. 3).

### Comparison between domains

The correlation between resilience across different domains was generally weak, with moderate correlations observed only between mobility and usual activities, as well as between self-care and usual activities (Table 3).

**Table 3:**
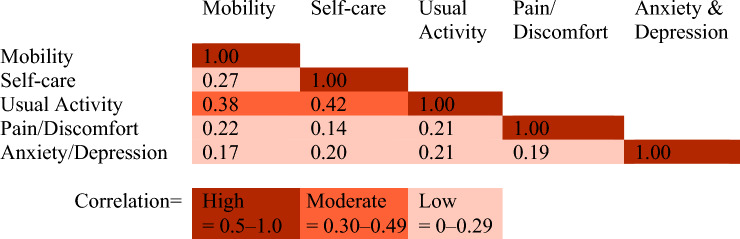
Correlation between resilience in the five EQ5D domains

Although direct comparable comparison is limited by differences in study populations, the only factors consistently associated with maintaining pre-fracture levels across all domains were taking no FRIDS and assisted walking ability, which was negatively associated with resilience (Fig. 3).

## Discussion

Despite the anticipated overall decline in health-related quality of life (HRQoL) 4 months after the hip fracture, variations across different EQ5D domains were observed. It was common for participants to report unfavorable levels in the domains of Mobility, Usual activities, and Pain/Discomfort even before the fracture occurred. The exception was Anxiety/Depression where patients reported relatively favorable levels prior to the fracture. The proportion of individuals who maintained (or improved) their EQ5D levels (indicating resilience in HRQoL) also varied across the domains, ranging from 36 to 77%, with Mobility being the most affected domain and Anxiety/Depression the least affected. In general, hip fracture patients were still affected in their HRQoL related to physical function but not so much mentally. Indeed, we showed that the correlation between being resilient in the different domains was low.

When examining factors associated with resilience in the different EQ5D domains, only a low medication burden (having no FRIDs) was consistently associated with maintained levels across all domains. Conversely, assisted walking ability prior to the fracture was negatively associated with resilience in all domains. This suggests that the global EQ5D might obscure distinct resilience profiles. However, both deprescribing medications and physical training to maintain walking ability are feasible interventions of modifiable factors that can be a part of a secondary prevention program [25, 26].

Women had a smaller average decrease in global HRQoL, and regression analyses indicated that being woman was associated with greater resilient in most domains compared to men. Similarly, previous research has shown that women tend to survive and manage diseases and disabilities to a greater extent than men, who not only have higher mortality rates after hip fractures but also tend to report lower HRQoL afterward [27, 28].

Mobility, Self-care, and Usual activities exhibited similar patterns both before and after the fracture. These three domains also showed comparable associations with related sociodemographic, medical, and acute factors. It is well established that physical function and mobility are closely correlated with the ability to perform daily activities and self-care tasks [29, 30]. It is also anticipated that self-rated mobility and usual activities are affected by a hip fracture, an acute physical injury [31]. The domain least affected both pre- and post-fracture was Anxiety/Depression, contradicting previous research, showing that mental health is affected by an injury or a fracture among older adults [32]. It might be more difficult to answer truthfully about one’s mental health for an older population, compared to somatic health, due to stigma [33]. It is also possible that one single question is too crude to catch the complexity of mental health, in comparison to the more concrete domains such as mobility and usual activities. In addition, considering the links between pain and depression among older adults [34], it is somewhat surprising that the correlation between Pain/Discomfort and Anxiety/Depression was low.

Almost 70% of the study population reported some sort of problems with pain already before the fracture. This is higher than other reports, for example compared to a Swedish study within the same age group that shows a prevalence of chronic pain with less than 40% [35]. However, a recent study among the oldest old (+ 85), shows similar prevalence as our study [36]. For Pain/Discomfort, the associations with sociodemographic, medical, and acute factors exhibited different patterns compared to the other domains. Given the high prevalence of pain also prior to the fracture, it is challenging to determine the extent to which the pain reported after the hip fracture is attributable to the injury itself or to a worsening of pre-existing pain issues. Resilience in the Pain/Discomfort domain was associated with having dementia and residing in a care home. This could suggest that improved pain management is facilitated by the availability of healthcare resources. However, it may also reflect the challenges individuals with dementia face in expressing their pain and the difficulties that their caregivers encounter in interpreting their pain-related expressions [37]. Also, some of the study participants with a dementia diagnosis had a next-of-kin reporting for them, causing uncertainty for the less visible domains, such as pain and depression.

The only factor consistently associated with resilience in HRQoL across all domains was absence of FRIDs. It is well established that FRIDs are strongly linked to the risk of falls and related injuries [38]. The concept of FRIDs is developed to assess risk for a fall, not the prognosis after it. However, this study indicates that FRIDs can be a good tool to assess prognosis after a fall injury as well. It is conceivable that these medications create a double burden for older adults, stemming both from the underlying conditions being treated and from the adverse effects of the medications themselves. Consequently, the use of FRIDs may serve as a more accurate measure of health vulnerability than traditional assessments of co-morbidity.

It should be noted that EQ5D is self-reported, and the baseline measure point is collected in conjunction with the hospital stay due to the hip fracture and surgery. Even if the individuals were asked about their status the previous week, their current stressful situation might influence their answers. Also, in situations where the questions were answered by a proxy, the uncertainty is even bigger, especially for abstract factors such as pain/discomfort and mental wellbeing. Previous estimates in SHR show that about 25% of answers are given by a proxy and that this is closely related to cognitive function. However, our study population consists of individuals with non-missing data and that survived at least 4 months after the fracture, so the proportion of proxy is likely lower in this study. Since one of the aims of this study was to investigate which characteristics were associated with maintaining one’s EQ5D, individuals reporting poor level in the domains already at baseline were excluded from the physical resilience part of the study. This is, because of the floor effect of the EQ5D, where individuals with low EQ5D would be defined as physical resilient to a higher degree. It is easier to maintain a lower level of HRQoL compared to the highest level, as apparent in the regression analysis where worse pre-fracture EQ5D domain status was associated with being resilient. Hence, the study population is not a representation of the general hip fracture population, but rather a healthier one. Whether it is optimal to measure outcomes after a hip fracture already after 4 months is debated. However, previous studies show that the decline in HRQoL is manifested 1 month after the fracture and remains several years [39, 40].

We observed a mean EQ5D Index of 0.72 pre-fracture. This is lower than what has been reported for general populations of the same age (scores between 0.84 and 0.90) [41]. This shows that older individuals that endure a hip fracture are, already prior to the fracture, a vulnerable group with poorer health and worse prerequisites to maintain independence and quality of life after the fracture, as previous shown by us and others [42]. The need for both primary and secondary prevention and a person-centered approach for this patient group is essential [43].

In conclusion, this study demonstrated that even though hip fracture patients face an overall decline in health-related quality of life (HRQoL), a substantial proportion remain resilient 4 months after the fracture. However, we found a large variability in resilience between the different EQ5D domains, with the domains related to physical function most affected and mental health the least affected. Factors associated with resilience in the different domains also differed, suggesting that a global EQ5D Index might masque different resilience profiles. Still, there are factors associated with resilience across all EQ5D domains, namely not taking FRIDs (high-risk medications) and pre-fracture walking ability. These are modifiable factors that are suitable targets for secondary prevention.

## Supplementary Information

Below is the link to the electronic supplementary material.

Supplementary file1 (DOCX 365 KB)

## Data Availability

Due to the GDPR law in Sweden, the pseudo-anonymized personal data in this study are based on cannot be shared publicly. Access to the data and the codes for data analyses can be permitted to external researchers after established collaborative agreement and ethical approvals. Contact the corresponding author for questions about data sharing (SE).
