# Supplementary material for: The impact of hip fracture on resilience in health-related quality of life: a cohort study
Source: Eur Geriatr Med. 2025 Apr 29;16(3):963–73. doi: 10.1007/s41999-025-01213-z (PMC12174284; doi:10.1007/s41999-025-01213-z)
Supplement: Supplementary file 1 — Supplementary file1 (DOCX 365 KB) [file 41999_2025_1213_MOESM1_ESM.docx]

**Supplementary material**


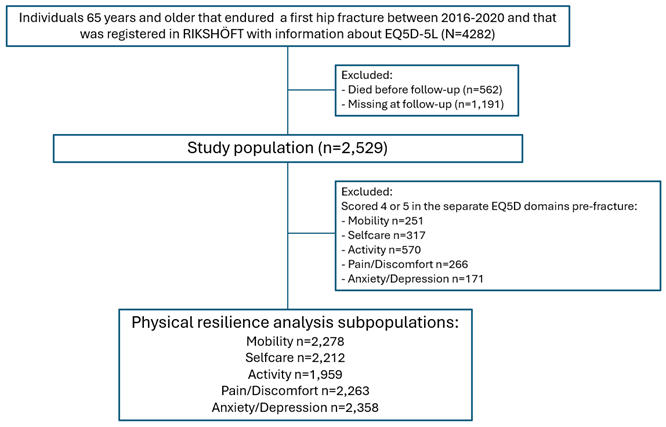


**Appendix figure 1**. Flowchart of the study population.

**Appendix Table 1**. Comparison of baseline characteristics of the study population, individuals that dies before follow-up and individuals with missing follow-up data.

|  | Study population, n=2,529 | Dead within 4 months, n=562 | Missing at FU, n=1,191 |
| --- | --- | --- | --- |
| **Sex** |  |  |  |
| Women | 1,752 (69.3) | 322 (57.3) | 831 (69.8) |
| Men | 777 (30.7) | 240 (42.7) | 360 (30.2) |
| **Age** |  |  |  |
| 65-79 years | 860 (34.0) | 74 (13.2) | 423 (35.5) |
| 80-89 years | 1,171 (46.3) | 259 (46.1) | 561 (47.1) |
| 90+ years | 498 (19.7) | 229 (40.8) | 207 (17.4) |
| **Cohabitation** |  |  |  |
| Co-living | 931 (36.8) | 134 (23.8) | 431 (36.2) |
| Living alone | 1,129 (44.6) | 173 (30.8) | 624 (52.4) |
| Care home | 469 (18.5) | 255 (45.4) | 136 (11.4) |
| **Education** |  |  |  |
| Primary | 1,213 (48.0) | 304 (54.1) | 601 (50.5) |
| Secondary | 882 (34.9) | 134 (23.8) | 396 (33.2) |
| University | 434 (17.2) | 255 (45.4) | 194 (16.3) |
| **ASA score** |  |  |  |
| 1-2 | 1,147 (45.4) | 117 (20.9) | 544 (46.1) |
| 3-5 | 1,379 (54.6) | 443 (79.1) | 635 (53.9) |
| **Fracture type** |  |  |  |
| Intracapsular | 1,384 (54.7) | 297 (52.9) | 641 (53.8) |
| Pertrochanteric | 937 (37.1) | 225 (40.0) | 466 (39.1) |
| Subtrochanteric | 208 (8.2) | 40 (7.1) | 84 (7.1) |
| **Surgery method** |  |  |  |
| Less invasive | 870 (34.4) | 201 (35.8) | 465 (39.0) |
| More invasive | 1,659 (65.6) | 361 (64.2) | 726 (61.0) |
| **Medications** |  |  |  |
| FRIDs 0 | 360 (14.2) | 46 (8.2) | 169 (14.2) |
| FRIDs 1-2 | 922 (36.5) | 171 (30.4) | 435 (36.5) |
| FRIDs 3+ | 1,247 (49.3) | 345 (61.4) | 587 (49.3) |
| **Dementia** | 377 (14.9) | 222 (39.5) | 109 (9.2) |
| **Depression** | 106 (4.2) | 23 (4.1) | 42 (3.5) |
| **Walking ability, pre-fracture** | |  |  |
| High | 1582 (62.5) | 161 (28.8) | 833 (70.1) |
| Intermediate | 808 (32.0) | 316 (56.4) | 300 (25.3) |
| Low | 139 (5.5) | 83 (14.8) | 55 (4.6 ) |

**Appendix table 2**. EQ5D Index means of the study population, individuals that died before follow-up, and with missing follow-up information (a score of 0 being the worse possible and 1 being optimal).

|  | Pre-fracture | | |
| --- | --- | --- | --- |
|  | Study population | Dead within 4 months | Missing at FU |
| **All** | 0.72 | 0.53 | 0.75 |
| **Sex** |  |  |  |
| Women | 0.73 | 0.51 | 0.74 |
| Men | 0.70 | 0.56 | 0.76 |
| **Age** |  |  |  |
| 65-79 | 0.76 | 0.50 | 0.77 |
| 80-89 | 0.71 | 0.53 | 0.73 |
| 90+ | 0.65 | 0.55 | 0.74 |
| **Education** |  |  |  |
| Primary | 0.71 | 0.54 | 0.73 |
| Secondary | 0.72 | 0.53 | 0.76 |
| University | 0.73 | 0.50 | 0.78 |
| **Living status** |  |  |  |
| Cohabiting | 0.78 | 0.60 | 0.80 |
| Alone | 0.77 | 0.65 | 0.76 |
| Care home | 0.46 | 0.23 | 0.49 |
| **ASA** |  |  |  |
| 1-2 | 0.79 | 0.58 | 0.80 |
| 3-5 | 0.66 | 0.52 | 0.70 |
| **Walking ability** |  |  |  |
| High | 0.82 | 0.73 | 0.82 |
| Intermediate | 0.59 | 0.49 | 0.62 |
| Low | 0.30 | 0.31 | 0.32 |


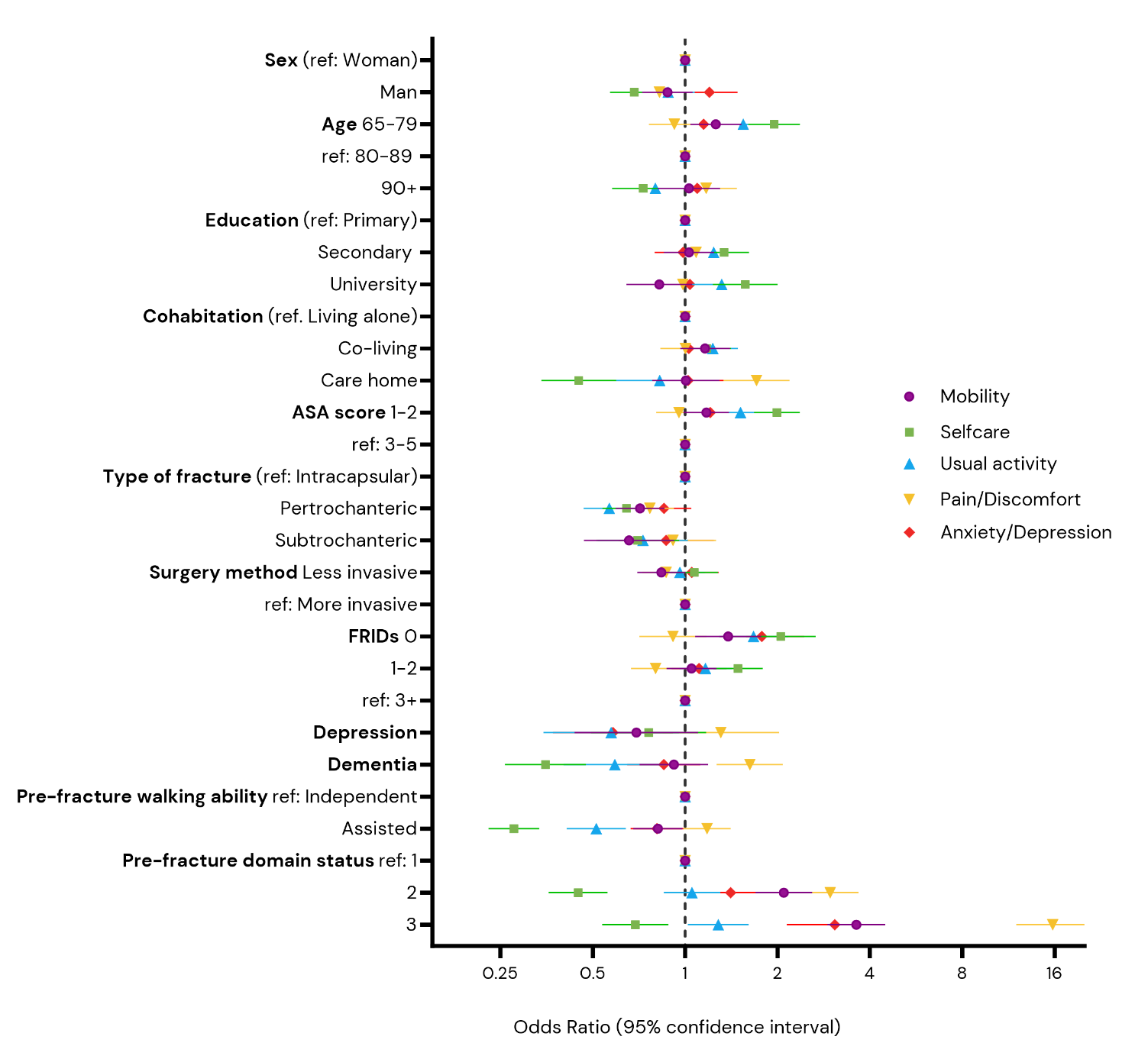


**Appendix figure 2**. Association between sociodemographic, fracture related- and medical factors and maintaining EQ5D level 4 months after a hip fracture, per EQ5D domain. Crude model.
